# Impact of Apple Pulp on Textural Characteristics, Microstructure, Volatile Profile, and Sensory Acceptance of Yogurts

**DOI:** 10.3390/foods14142453

**Published:** 2025-07-12

**Authors:** Dimitra Dimitrellou, Thomas Moschakis, Panagiotis Kandylis

**Affiliations:** 1 Department of Food Science and Technology, Ionian University, 28100 Argostoli, Greece; 2 Department of Food Science and Technology, School of Agriculture, Aristotle University of Thessaloniki, 54124 Thessaloniki, Greece; tmoschak@agro.auth.gr

**Keywords:** yogurt, apple, texture analysis, sensory evaluation, aroma profile, microstructure, GC/MS

## Abstract

Fresh apple pulp from the Granny Smith variety was used at different levels (5–15% *w*/*w*) for yogurt production. Color, texture, microstructure, aroma, and sensory analyses were used to evaluate the effect of the apple pulp on the main characteristics of yogurt. Yogurts with apple pulp presented a lower brightness (L*) and an increased redness (a*) and yellowness (b*), which were significantly affected by the apple pulp concentration. The texture analysis revealed an improved consistency and reduced syneresis, leading to a creamier and more stable product. The aroma profile of yogurts was enriched, presenting higher ester contents. Confocal laser scanning microscopy showed that the incorporation of modest quantities of apple pulp resulted in the formation of initially denser networks, while at elevated levels, an enhanced microscopic phase separation occurred. A 5% apple pulp addition achieved a balance between enhancing flavor and texture retention while maintaining high overall acceptability, as was also confirmed by the sensory evaluation.

## 1. Introduction

Yogurt is a popular worldwide fermented dairy product, having ancient origins and becoming part of the diet of Ancient Greeks and peoples of Mesopotamia, the Middle East, and Central Asia [1]. Traditionally, yogurt is produced using two specific bacterial cultures, namely *Lactobacillus delbrueckii* ssp. *bulgaricus* and *Streptococcus thermophilus*, that generate lactic acid, resulting in milk solidification, providing its unique creamy texture and tangy flavor. Yogurt has been characterized as a significant functional dairy product, which holds a large share of the global dairy market and is known for its rich nutritional profile, which includes protein, fat, minerals (calcium, potassium, etc.), and vitamins (especially B complex) [2]. The regular consumption of yogurt is associated with numerous health benefits, and it is characterized as suitable for the diet of individuals with lactose intolerance, hypertension, diabetes, and cardiovascular conditions [2,3].

Nowadays, there is a growing demand by consumers for healthier products and balanced nutrition. Based on that, the yogurt industry faces novel challenges like reducing sugar contents; improving the appearance, flavor, and texture; enhancing nutritional values; and extending the product’s shelf life [4]. Following that trend, there are several studies available in the literature regarding the fortification of yogurts with grapes, fruits, vegetables, cereals, and plant extracts in order to further improve their functional characteristics [5,6,7]. However, the incorporation of such products is expected to alter the textural and sensorial characteristics of yogurts, affecting consumer acceptance. Therefore, it is also necessary to conduct a comprehensive evaluation of the sensorial characteristics of these fortified yogurts.

Among several fruits that have been incorporated in yogurts, apple and its derivatives have been evaluated. Several studies are available in the literature regarding yogurts with incorporated apple pomace, in various forms like flour, powder, syrup, etc. [8,9,10,11,12], and apple fiber [13]. The existing literature includes studies on the addition of apple pulp to yogurt; however, these investigations have been limited to assessing only the primary physicochemical characteristics [14,15,16]. The effect on textural characteristics is important since it affects the purchasing power of the yogurt. In a similar study but with apple pomace, its addition affected the yogurt’s texture, rheological behavior, and microstructure [17]. More specifically, in yogurts with apple pomace, gelation began earlier, while the gel firmness and cohesiveness was increased during storage. Confocal imaging showed that at 0.5% apple pomace, the yogurt exhibited a uniform, well-connected microstructure with thick casein strands, suggesting this concentration may be optimal for achieving a strong, stable yogurt gel. Previous studies revealed that the addition of fruit pulps, especially at concentrations up to 10%, does not seem to significantly affect the textural characteristics of yogurts [18,19,20]. Only, a decrease in hardness was observed in the case of jujube pulp [20] and a decrease in cohesiveness in the case of sea buckthorn, elderberry, and Sloe Berry yogurts [19]. However, to the best of our knowledge, there are no available studies evaluating the effect of an apple pulp addition on the textural and aroma characteristics of yogurts.

Therefore, the present study aims to respond in this gap of the literature by evaluating the effect of the addition of apple pulp at different concentrations on yogurts’ textural characteristics, microstructure, aroma, and color.

## 2. Materials and Methods

### 2.1. Materials

For the purposes of this study, pasteurized and homogenized cow’s milk (Olympos, Larisa, Greece) was utilized, characterized by a composition of 3.7 g fat, 4.7 g carbohydrates, and 3.4 g protein per 100 mL and total solids 12.5%. Skim milk powder (Regilait, Mâcon, France), with a composition of 0.8 g fat, 52 g carbohydrates, and 36 g protein per 100 g, was used. Apple samples of the Granny Smith variety, cultivated in Greece, were procured from a local retailer. Yogurt fermentation was carried out using a thermophilic starter culture, CH-1 (Chr. Hansen, Hørsholm, Denmark).

### 2.2. Apple Pulp and Yogurt Production

Apples at full ripeness and suitable for consumption were washed using warm water at 45 °C, followed by manual removal of seeds and peels. The resulting apple pulp was prepared using a hand blender (Severin Elektrogeräte GmbH, Sundern, Germany) and subsequently pasteurized at 79 °C for 69 s, following the protocol described by Senadeera et al. [21]. The pasteurized pulp (total solids 14.80 ± 0.30%, measured using an AMB 310 moisture balance, Adam Equipment Co. Ltd., Milton Keynes, UK) was then incorporated into the yogurt production process.

Yogurt preparation was conducted according to the method described by Dimitrellou et al. [22]. Apple pulp was incorporated at concentrations of 0%, 5%, 10%, and 15% (*w*/*w*) in milk containing 3% (*w*/*w*) skim milk powder (total solids 15.14%), 10 min prior to the inoculation with the starter culture. Fermentation was carried out at 42 °C until the pH reached 4.65–4.70 (fermentation time 219 ± 9 min), after which the samples were stored at 4 °C for up to 28 days. The pH was determined using a FC200B pH electrode (Hanna instruments, Woonsocket, RI, USA).

### 2.3. Analyses

#### 2.3.1. Color Analysis

Color of yogurts was measured after 1 day of storage using a colorimeter (CR-410 Chroma Meter, Konica Minolta Inc., Osaka, Japan) according to Dimitrellou et al. [22].

#### 2.3.2. Texture Analysis

A texture analyzer (IMADA Inc., Northbrook, IL, USA) was used to determine the Texture Profile Analysis (TPA) of the yogurt samples during the storage period (days 1, 14, and 28 of storage). A compression test was performed on the yogurt samples (set yogurt samples prepared in 100 mL cups) with the following test parameters: cylinder probe diameter 20 mm, test speed 1.0 mm/s, penetration distance 25 mm, and a 5 kg load cell. Each yogurt sample was subjected to two successive compressions to obtain the force (N)–time (s) curve [20]. The parameters calculated from the Force Recorder Professional (FRTS Ver.) version 1.03 (IMADA Inc., Northbrook, IL, USA) software were hardness, cohesiveness, springiness, gumminess, and chewiness. Each yogurt sample was analyzed in triplicate.

#### 2.3.3. Microstructure of Yogurt

Approximately 5 mL of yogurt samples after 1 day of storage and 20 μL of 0.01% (*w*/*v*) Nile Blue (stained proteins) and Nile Red (stained the oil phase) solutions were combined thoroughly in a small beaker [23]. Subsequently, 2 mL of the mixture was transferred to a glass bottom dish with a thickness of 0.17 mm (WillCo Wells BV, Amsterdam, The Netherlands). The examination of their microstructure was conducted using a Leica TCS SP5 II confocal laser scanning microscope (CLSM), mounted on a Leica Model DM 6000 B inverted microscope (Leica Microsystems, Wetzlar, Germany) operating in the fluorescence mode with a 63 × oil-immersion objective and a numerical aperture of 1.40. To avoid hydrodynamic interactions with the coverslip, image scanning was performed at a depth of approximately 20–30 μm below the coverslip. Excitation of the sample fluorescence was achieved through the use of a 488 nm Argon laser and the 633 nm line of a red HeNe laser. Image dimensions were adjusted to 512 × 512 pixels in the x–y plane. The signal from the samples was gathered, and an image of the average of eight scans was produced. All measurements were performed at 25 °C.

#### 2.3.4. GC/MS Analysis

For the evaluation of volatile compounds, yogurt samples (7 g; homogenized samples were prepared in accordance with the method described in ISO 11869:2012 [24]) after 14 days of storage were subjected to headspace solid-phase microextraction (HS-SPME) GC/MS analysis, using the procedure described in a previous study [25]. Yogurt samples were transferred into 20 mL headspace vials sealed with Teflon-lined septa and aluminum crimp caps. Volatile compounds were extracted using a SPME fiber (2 cm length, coated with a 50/30 µm Divinylbenzene/Carboxen/polydimethylsiloxane [DVB/CAR/PDMS] stationary phase, Supelco Inc., Bellefonte, PA, USA) introduced through the septum. The vials were incubated at 80 °C for 30 min to allow headspace equilibration and analyte adsorption onto the fiber. Following extraction, the fiber was immediately inserted into the gas chromatograph (GC) injection port, maintained at 280 °C, for thermal desorption of analytes under splitless mode for 2 min. Separation was carried out on a Supelco CO Wax-10 capillary column (60 m length × 0.32 mm internal diameter × 0.25 µm film thickness) (Supelco Inc., Bellefonte, PA, USA). The oven temperature was programmed as follows: initial hold at 35 °C for 3 min, then increased at 5 °C/min to 110 °C, followed by a ramp of 10 °C/min to a final temperature of 240 °C. Helium was used as the carrier gas at a constant flow rate of 1.5 mL/min. Detection was performed using a GC-MS system operating in electron impact ionization mode at 70 eV. The identification was carried out as follows: (1) by comparing the retention times and mass spectra of volatiles to those of standard compounds (ethyl butanoate, ethyl octanoate, ethyl decanoate, octanoic acid, decanoic acid, 3-methyl-1-butanol, 1-hexanol, 1-octanol, 2-heptanol, 2-nonanol, 2-ethyl-1-hexanol (Sigma–Aldrich, Poole, UK)), (2) by mass spectra obtained from NIST107, NIST21, and SZTERP libraries, and (3) by determining Kovats’ retention indexes and comparing with those reported in the literature (in all cases the minimum similarity percentage provided was established at 80%). Semi-quantification of volatile compounds was carried out using methyl octanoate (Sigma–Aldrich, Poole, UK) as an internal standard (at concentration of 6.3 μg/kg of yogurt). The volatile compounds were semi-quantified by dividing the peak areas of the compounds of interest by the peak area of the internal standard and multiplying this ratio by the concentration of the internal standard (expressed as μg/kg of yogurt).

#### 2.3.5. Sensory Analysis

A sensory evaluation was conducted for all yogurt samples 5 days after production with 69 untrained panelists (30.4% men and 69.6% women, 21–46 years old). All the panelists were staff members and undergraduate students of the Department of Food Science and Technology. All panelists were very familiar with yogurt characteristics, with a regular yogurt consumption. Moreover, three background questions were asked in order to determine their consumption frequency of yogurt and to evaluate their knowledge of the health benefits of yogurt and their purchase intention (order of preference) of the tested yogurts [26]. A nine-point hedonic scale was utilized, evaluating selected quality attributes. Sensory analysis consisted of appearance (color, viscosity), aroma (sweet, sour, fruity), flavor (sweet, sour, fruity, bitter), mouthfeel (mouthcoat, smoothness, astringency), aftertaste, and overall acceptability (aroma, taste, appearance, and general acceptance) [26,27,28]. The samples (set yogurt sample per participant) were randomly labeled and served to panelists. Water and crackers (without sugar and salt) (Elite, ELBISCO S.A., Pikermi, Greece) were served in order to clean the panelists’ palate. All the sensory attributes were previously explained to the panelists. Panelists were permitted to taste the samples prior to the commencement of the testing procedure, and they were informed of the type of product being tested. All panelists participated voluntarily and signed an informed consent form, which was reviewed and approved by the Research Ethics and Deontology Committee of Ionian University (2535/18-07-2024 6th committee meeting).

### 2.4. Statistical Analysis

All experiments were performed in triplicate, and duplicate samples were obtained for each type of analysis. Statistical evaluation of the data was carried out using analysis of variance (ANOVA), followed by Tukey’s Honest Significant Difference (HSD) test for post hoc comparisons. Coefficients, ANOVA, and principal component analysis (PCA) were conducted using Statistica software, version 12.0 (StatSoft Inc., Tulsa, OK, USA).

## 3. Results and Discussion

### 3.1. Color Characteristics of Yogurts

Color is an important characteristic for the food industry and especially for products like yogurts, where the appearance influences the consumer perception and acceptance. It is well-known that the addition of products like apple derivatives influences the color parameters of yogurts. These changes are highly dependent on the type and amount of the apple derivative used. The same applies for apple pulp, as reported in the present study.

**Figure 1:**
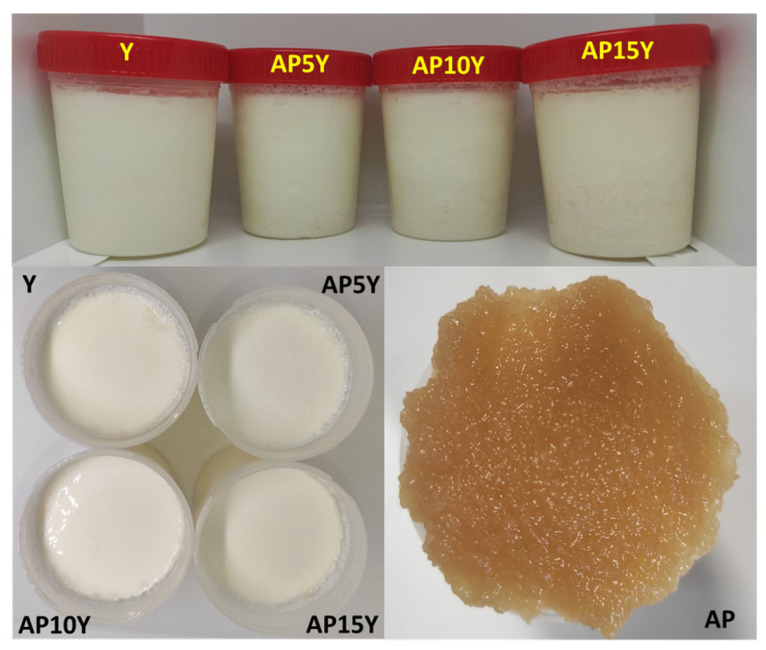
Photographs of the yogurts after 1 day of storage and the apple pulp before the addition to milk (Y: yogurt without apple pulp; AP5Y, AP10Y, and AP15Y: yogurts with apple pulp 5, 10, and 15% *w/w,* respectively; AP: apple pulp).

The incorporation of apple pulp significantly decreased (*p* < 0.05) the L* value as the amount of pulp increased, making yogurts appear darker (Table 1, Figure 1). This effect is due to the inherent color of the apple pulp (L* = 40.2 ± 0.0) being darker than the control yogurt (L* = 92.6 ± 1.1). Similar results for L* values were reported for yogurts fortified with apple derivatives. More specifically, as the amount of the apple pomace powder (1% to 3%, freeze-dried) [10], apple pomace syrup [11], and apple pomace powder (0.2% to 1.0%, oven-dried) [9] increased, the lightness (L*) decreased. This tendency for darker yogurts may be attributed to the enzymatic browning of the apple pulp, which also explains its low L* value (40.2).

The addition of apple pulp also affected the a* and b* values of yogurts. Both values were increased after the addition of the apple pulp, resulting in a more intense red (higher a* values) and yellow coloration (higher b* values). More specifically, the increased content of apple pulp resulted in significantly (*p* < 0.001) higher values of a* and b*. These changes in a* and b* values may be attributed to the polyphenolic compounds present in apple pulp. Other researchers also reported a redder and yellowish color of yogurt fortified with apple derivatives [9,10,11].

The chroma (C), a representation of the saturation or intensity of the color, of the produced yogurts increased (10.8 in Y to 12.7 in AP15Y), indicating a more intense color due to the addition of the apple pulp. The hue angle decreased significantly (*p* < 0.001) as the apple pulp increased (111.2 in Y to 96.6 in AP15Y), indicating a tendency towards the red–yellow spectrum.

### 3.2. Texture Analysis of Yogurts with Apple Pulp

Knowing the texture of yogurt samples is an important aspect of quality assessment [9]. Texture depends on many factors, mainly related to the yogurt composition [8]. Table 2 presents the texture parameters of the produced yogurts.

One of the most important textural parameters of yogurt is hardness. Hardness, also called firmness, is the force required to cause deformation [29]. It is affected by the total solids contained in yogurt samples and corresponds to the yogurt coagulation [20]. The addition of apple pulp had no effect (*p* > 0.05) on the hardness of yogurts. Furthermore, a slight but not significant decrease (*p* > 0.05) was observed with storage time. These results are in accordance with other studies using pulps (cupuassu and jujube) or purees (sea buckthorn, elderberry, and sloe fruit) in yogurt production [18,19,20]. However, in studies using constitutes in the form of powder, an increase in firmness was detected that was attributed to the increased total solid content [9,10]. The starter culture composition and especially the content of *L. delbrueckii* ssp. *bulgaricus* have been correlated with the values of hardness in fermented milks [30]. The content of the total solids and, in particular, the protein content and the type of protein [31] also exert an influence on hardness; however, in the present study, the presence of apple pulp did not affect these parameters. In the present study, apple pulp had a similar total solid content (14.80%) to the milk (15.14%), and therefore its addition did not significantly affect the total solid content of the final product and the subsequent hardness. Furthermore, the pH value of all yogurts was the same after the production, and therefore it is not expected to affect these properties.

Cohesiveness is another key texture characteristic of yogurt, corresponding to the force required to break the inner bond links of the product [29] and the ability of the sample to hold together rather than spreading across the tongue and the surfaces of the mouth [32]. A tendency for an increased cohesiveness with an increasing the apple pulp was observed (*p* < 0.01). This can be attributed to the creation of stronger structures in yogurts containing apple pulp. The presence of fibers can reduce serum separation [33], so the fiber content of the apple may reduce the syneresis, leading to the increased cohesion of these yogurts. The incorporation of the apple pomace flour/powder in yogurts also led to an increase in cohesiveness values [8,9,10]. The cohesiveness values of all yogurt samples showed a slight but not significant (*p* > 0.05) increase during the storage period. Similar results, with no significant differences in the cohesiveness during storage, were also observed in yogurts with *Melastoma dodecandrum Lour* fruit powder [34], with date palm coproducts [35], and with cupuassu pulp [18]. In general, the yogurt without apple pulp presented the lowest cohesiveness values, which is correlated with a smoother gel structure, as was also observed during the sensory evaluation in the present study [36].

Gumminess reflects the required mechanical work to compress the yogurt sample until it is ready to swallow [37]. It is considered as a defect that negatively affects the appearance and texture [29]. In the present study, neither the addition of the apple pulp nor the storage time significantly affected (*p* > 0.05) the gumminess of the yogurt. In previous studies, the addition of the apple pomace powder led to a decrease in gumminess [9], while the addition of flaxseed led to an increase [29].

Chewiness is the required energy to masticate in order to swallow the yogurt sample [37], and it is related to firmness, cohesiveness, and elasticity. The incorporation of apple pulp significantly (*p* < 0.01) increased chewiness levels, whereas the storage duration had no effect. These results are similar to a study adding flaxseed in yogurts [29]. The increased chewiness in yogurts with apple pulp may be attributed to the pulp’s viscosity, which may further enhance the yogurt’s structure, or to the presence of apple pulp particles requiring a greater effort for chewing.

Springiness refers to how quickly a yogurt sample returns to its original shape after the deforming force is removed. This characteristic is influenced by several factors, including heat treatments, protein interactions, flexibility, and the degree to which the proteins unfold [38]. Springiness indicates the texture integrity of the yogurt, and the addition of apple pulp in yogurt may increase the texture integrity. The addition of apple pulp significantly affected the springiness, leading to higher values, especially at higher concentrations (AP10Y and AP15Y). A slight but not significant increase (*p* > 0.05) was observed with an increasing storage time. A similar trend was reported in yogurts with apple pomace powder [9] and flaxseed [29]. However, when jujube pulp was added no significant differences were observed [20].

### 3.3. Microstructure of Yogurts with Apple Pulp

**Figure 2:**
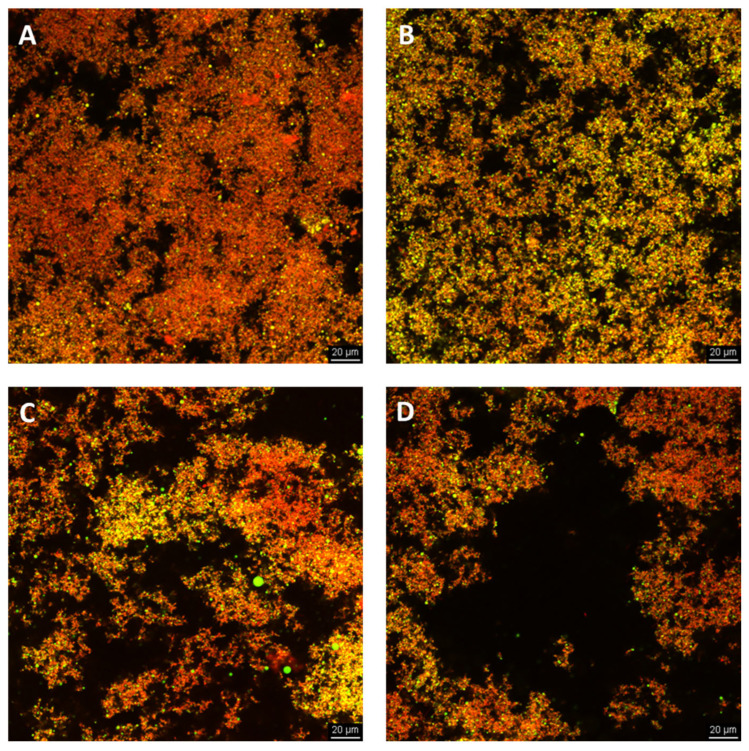
Confocal micrographs of yogurts (after 1 day of storage): (**A**) yogurt without apple pulp (Y); (**B**) yogurt containing 5% apple pulp (AP5Y); (**C**) yogurt containing 10% apple pulp (AP10Y); and (**D**) yogurt containing 15% apple pulp (AP15Y). (The green color indicates the oil phase, while the red color corresponds to the protein phase. The adsorbed protein surrounding the oil droplets appears yellow in the overlay image. These images are presented for a qualitative visual comparison only; no quantitative image analysis was performed. Scale bars: 20 μm).

Confocal laser scanning microscopy enabled the detailed visualization of the yogurt’s microstructure, revealing how the apple pulp integrates with the protein network. Figure 2 shows confocal microscopy images of yogurts after 1 day of storage. These images are of a qualitative nature and provide visual, comparative insights into the differences between the samples. The confocal micrographs were used to illustrate general trends in the protein and fat phase distributions. Notably, no discernible alterations in the microstructure were observed over a four-week storage period, indicating the structural stability of the yogurt matrix during refrigerated storage.

Yogurts with a low apple pulp (Figure 2B) content showed a continuous protein network surrounding oil droplets, with few large pores (voids). These pores, which cause phase separation on a micro-level, may enhance the yogurt matrix by creating a more compact and interconnected structure and a denser protein network. Increasing apple pulp contents led to larger pores and unevenly distributed the apple pulp within the protein network. However, as the apple pulp content increased, extensive phase separation on a micro-level and an uneven distribution were observed (Figure 2C,D), which may potentially lead to macroscopic phase separation, which may affect the overall texture and mouthfeel of the yogurt.

The texture analysis showed that the addition of apple pulp generally improved the cohesiveness, springiness, and chewiness of the yogurt, particularly at higher concentrations. These effects are linked to the apple pulp’s fiber content, which can stabilize the yogurt matrix by reducing syneresis and improving the water-holding capacity. The effect of the apple pulp on the yogurt hardness is consistent with the microstructural observations. The incorporation of modest quantities of apple pulp resulted in the formation of initially denser networks. However, at elevated levels of apple pulp, enhanced microscopic phase separation led to macroscopic phase separation, which subsequently resulted in a reduction in hardness.

### 3.4. Volatile Compounds Based on HS-SPME-GC/MS Analysis of Yogurts

#### 3.4.1. Esters

In the yogurt with no apple fiber (Y) only two esters were detected, namely ethyl acetate and ethyl butanoate, and were in low concentrations. This is in accordance with previous studies using the same starter culture [39], and it was expected since the presence of esters in yogurts is known to correlate with extensive storage [40]. The addition of apple pulp increased the ester content both in terms of qualitative and quantitative aspects (Table 3). Esters are the most important group of aroma compounds in apples, significantly influencing the fruity and sweet notes of their flavor. Common esters such as butyl acetate and hexyl acetate are found in various apple cultivars, are responsible for the pleasant fruity aroma, and are considered as the major esters in apples and apple juices [41,42]. Other important esters found in relatively high concentrations in apple juices are ethyl butanoate and 2-methylbutyl acetate [41]. All these three esters (ethyl butanoate, butyl acetate, and 2-methylbutyl acetate) were detected in significantly higher concentrations in yogurts with apple pulp and especially in the yogurt with the highest content of apple pulp (AP15Y). These esters are responsible for fruity/sweet/banana (ethyl butanoate), pineapple (butyl acetate), and fruity (2-methylbutyl acetate) odors. However, the relatively high odor thresholds of butyl acetate (58 μg/L in water [43]) and 2-methylbutyl acetate (11 μg/L in water [44]) suggest that their contribution to the overall aroma of yogurts would be limited. In contrast, yogurts with apple pulp presented ethyl butanoate concentrations higher than the threshold value (0.9 μg/L in water [43]), revealing a more pronounced contribution to the fruity aroma.

#### 3.4.2. Alcohols

In all yogurts ethanol was detected. Apart from that, in the yogurt with no apple pulp (Y), four alcohols were detected, namely 1-hexanol, 2-ethyl-1-hexanol, 1-heptanol, and 2-nonanol, providing fruity, floral, herb/mushroom, and cucumber notes, respectively. The addition of apple pulp, as in the case of esters, significantly affected the number and the concentration of alcohols in yogurt samples. More specifically, the higher the content of the apple pulp, the higher the number and concentration of alcohols. The most abundant alcohols identified in apples include 1-butanol, 1-hexanol, 2-methyl-1-butanol, 3-methyl-1-butanol, and 2-ethyl-1-hexanol [42,45]. The majority of these alcohols were also detected in the yogurts of the present study, with the apple pulp changing the final aroma compared to the control. The presence of secondary alcohols such as 2-heptanol (AP10Y and AP15Y) and 2-nonanol (in all samples) was also observed in yogurts, typically resulting from the enzymatic reduction of the respective methyl ketones. Various dairy products, such as fermented milk, yogurt, and cheeses, often contain these alcohols [25,39,46,47].

#### 3.4.3. Acids

Organic acids were the group of compounds with the highest concentration. All yogurt samples exhibited similar concentrations of acids, and only in the case of butanoic acid were significantly higher concentrations reported in yogurts with apple pulp. This may be attributed to the fact that acids in apples are present at low concentrations and play a minor but also significant role in contributing to the overall aroma profile. In apple pulps, the primary acids identified are acetic acid and 2-methylbutanoic acid, contributing an acidic, vinegar-like aroma and cheesy and rancid odors, respectively [42].

#### 3.4.4. Aldehydes

Apart from acetaldehyde, which was identified in all yogurt samples at comparable concentrations and is considered as a key volatile in yogurts, only four aldehydes were detected, namely hexanal, heptanal, octanal, and nonanal. These were also the major aldehydes detected in previous studies with the same starter culture [39]. The aldehydes are responsible for green notes of immature fruit (hexanal and heptanal) and orange notes (octanal and nonanal). The addition of the apple pulp only affected the concentration of hexanal and nonanal, resulting in significantly higher concentrations in yogurts with apple pulp. Aldehydes are predominant in immature apples, providing green and fresh notes to the fruit’s aroma. The major aldehydes in apples include hexanal and (E)-2-hexenal, which are associated with green, grassy, and sometimes fatty notes [42]. These compounds are formed via the oxidation of fatty acids and are crucial during the early stages of apple development. As apples ripen, the concentration of aldehydes generally decreases [41], making way for the formation of esters and alcohols, and this explains the limited effect of the apple pulp in the aldehyde content of yogurts in the present study.

#### 3.4.5. Ketones

Ketones were detected in high concentrations, as is usual in fermented milks [46]. Acetone (sweet, fruity aroma) and methyl ketones, like 2-butanone, 2-heptanone, and 2-octanone (fruity, floral, and musty notes), were detected in all yogurts. The apple pulp did not affect the concentration of ketones compared to the control sample (Y), apart from 2-octanone. Ketones contribute significantly to the floral and sweet aspects of the apple aroma [41,42].

#### 3.4.6. Total Volatile Compounds

The addition of the apple pulp significantly affected (*p* < 0.05) the total concentration of all groups, leading to higher values in the yogurts with high apple pulp contents. The same was observed in the case of total volatile compounds concentrations. Finally, in the case of the qualitative analysis, the addition of apple pulp resulted in higher numbers of volatile compounds. More specifically, in Y 19 compounds, in AP5Y 24 compounds, and in AP10Y and AP15Y 27 compounds were detected.

#### 3.4.7. Principal Component Analysis Based on HS-SPME-GC/MS Analysis of Yogurts

**Figure 3:**
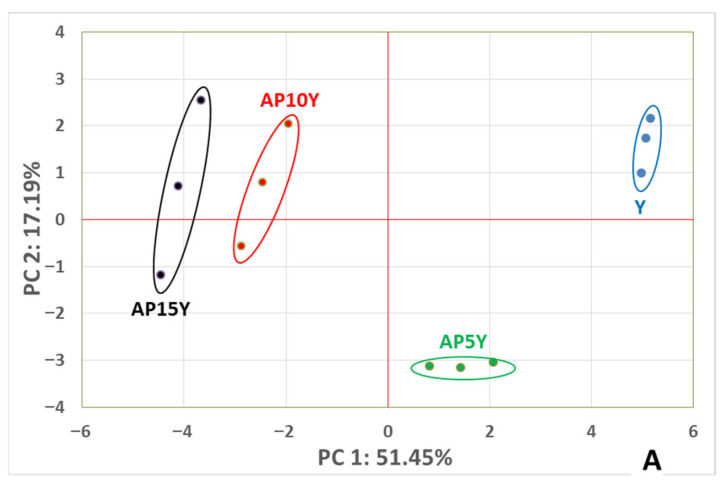

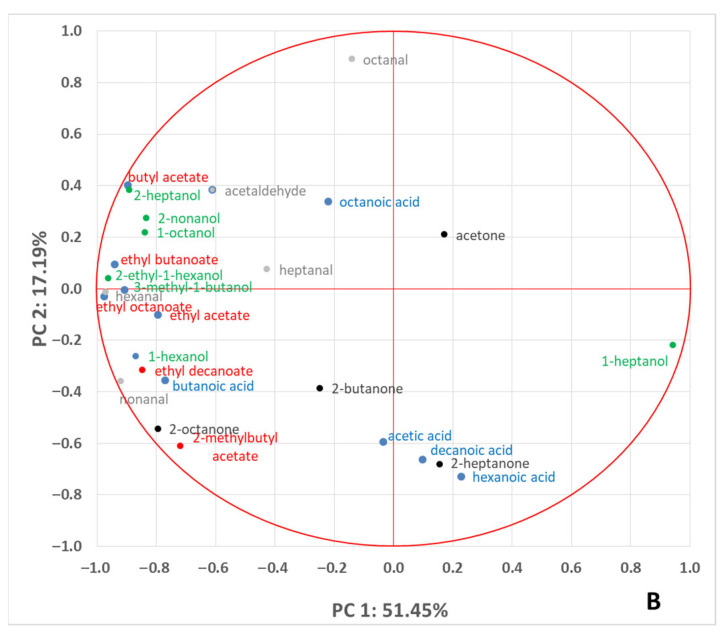
Principal component analysis (PCA) scores plot (**A**) and loadings plot (**B**) of volatile compounds detected in yogurts with apple pulp after 14 days of storage (Y: yogurt without apple pulp; AP5Y, AP10Y, and AP15Y: yogurts with apple pulp, 5, 10, 15% *w/w,* respectively. Compounds in red color are esters; in green color are alcohols; in blue color are acids; in gray color are aldehydes; and in black color are ketones).

The results of the HS-SPME-GC/MS analysis (all compounds presented in Table 3) were used to perform an unsupervised principal component analysis (PCA). As shown in Figure 3, PC1 interpreted 51.45% of the total variables and PC2 17.19%, so these principal components could explain more than 68% of the total variables. The addition of apple pulp significantly affected the volatile profile of the yogurts, since different groups of yogurt samples are made, which occupied independent spaces in the PCA model (Figure 3A). The yogurts with no apple pulp (Y) are placed at the upper right side of the plot, the AP5Y is at the bottom right, and the AP10Y and AP15Y are at the upper left side. The groups of different yogurts samples were distinguished efficiently, especially compared to Y. Among the yogurts with apple pulp, AP5Y was completely different compared to other samples, while AP10Y and AP15Y presented similar patterns since they are located in the same area at the upper left side of the plot. Therefore, it may be concluded that the addition of the apple pulp significantly affected the volatile composition of yogurts, especially up to the 10% addition. The further increase in the apple pulp up to 15% seems to have no notable impact.

The principal component analysis loadings plot (Figure 3B) confirmed the results of the scores plot—that the yogurt samples were grouped in three different groups. AP5Y correlated with acids like acetic, hexanoic, and decanoid, while AP10Y and AP15Y were correlated with more complicated aromas and volatile compounds from different groups like alcohols (2-heptanol, 2-nonanol, 1-octanol, 2-ethyl-hexanol, and 3-methyl-1-butanol), esters (butyl acetate and ethyl butanoate), and aldehydes (acetaldehyde and heptanal).

### 3.5. Sensory Analysis of Yogurts

**Figure 4:**
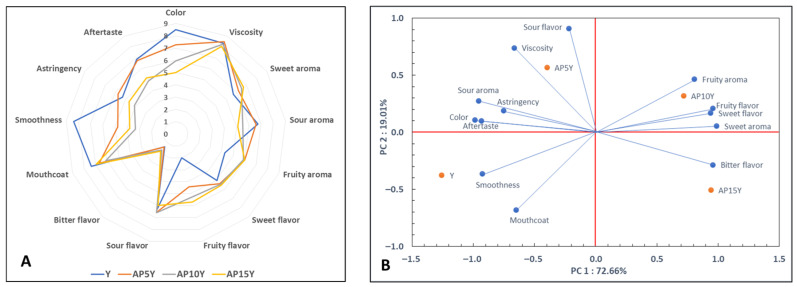
Sensory evaluation of yogurts after 5 days of storage (**A**) and sensory map using principal component analysis (PCA) (**B**) (Y: yogurt without apple pulp; AP5Y, AP10Y, and AP15Y: yogurts with apple pulp, 5, 10, 15% *w/w*, respectively).

A sensory analysis is necessary to measure the product quality and is crucial for dairy products [48]. It provides data directly related to consumer acceptance. In this study, the sensory characteristics of the yogurt with apple pulp were evaluated. The results of this evaluation are presented in Figure 4A. As expected, as the amount of added apple pulp increased, the color score lowered significantly (*p* < 0.05), as it deviated from the characteristic color of the milk observed in the control. The results also confirmed that samples with apple pulp received a slightly greater fruity aroma and a significantly greater fruity flavor (*p* < 0.05) compared to the control sample. As the apple pulp addition increased in yogurts, a lower score for the mouthfeel attribute was observed. A smoother texture was observed in the control sample (*p* < 0.05) due to the absence of the apple pulp particles.

The sensation caused by the apple pulp was determined to decrease the overall acceptability of the AP10Y and AP15Y samples (Table 4). This observation was also reported by Popescu et al. [9], where the yogurt containing the higher content of apple pomace received the lowest overall acceptability score. This was also supported by Senadeera et al. [21], where the highest texture score was recorded for the control yogurt compared to the yogurt containing Annona fruit pulp. The general characteristics of yogurts in their aroma and flavor were similar. The effect of adding apple pulp was significant (*p* < 0.05) in the case of the appearance, mouthfeel, and general acceptance. Similar scores were achieved for AP10Y and AP15Y, which were significantly lower than those of control (Y). Nevertheless, the AP5Y sample received similar ratings for the overall acceptability and purchase preference as the control. It can be concluded that the addition of particles to food matrices, like incorporating fruit in yogurt, alters the sensory perception and increases the consumer preference [49]. The analysis showed the favorability of the panelists towards the control and YA5 samples, among all the yogurt samples. In another study, the participants also preferred the control as well as the yogurt with the lowest concentration of pineapple pulp (0.1%) [36].

Figure 4B shows the PCA maps of the yogurts regarding sensory attributes and samples. The variation was mainly explained by the component PC1 (72.66%) followed by PC2 (19.01%), totaling 91.67% of the explanation. The total explanation can be considered relatively high for a study with untrained panelists, who have a greater difficulty in performing such tasks compared to trained panelists, and it is higher compared to other similar studies with untrained panelists [28].

The attributes of a fruity aroma, fruity flavor, sweet aroma, sweet flavor, and bitter flavor were positively correlated with PC1, while the attributes of a sour aroma, astringency, an aftertaste, and color were correlated with PC2 (Figure 4B). PC1 effectively distinguished the yogurt samples AP10Y and AP15Y (right of the axis) from samples AP5Y and Y (left of the axis). Thus, the yogurts with the highest apple pulp content (AP10Y and AP15Y) exhibited the most pronounced fruity and sweet aroma and flavor. In contrast, the yogurt with the lowest apple pulp content (AP5Y) and the yogurt without apple pulp (Y) displayed attributes related to the color, sour aroma, astringency, and aftertaste. Furthermore, AP5Y is notably distinguished by a sour flavor and viscosity attributes, while the control sample is associated with a smoothness and mouthcoat. The results of the sensory evaluation are in accordance with the findings of the GC/MS analysis of the yogurt samples. In particular, the increase, both from a qualitative and quantitative point of view, of the fruity esters of yogurts with apple pulp can be linked to the fruity aroma and flavor observed in these samples during the sensory evaluation.

## 4. Conclusions

The incorporation of apple pulp significantly influenced the volatile composition of the yogurts, particularly at concentrations up to 10%, and exhibited a more pronounced fruity aroma and flavor compared to the control sample. These findings suggest that while apple pulp can enhance certain sensory and textural attributes, its concentration must be carefully optimized to maintain consumer acceptability. An addition of 5% apple pulp appears to provide a balance between enhancing flavor and maintaining a favorable texture and overall acceptance. Future research should focus on further enhancing the flavor and appearance of yogurts, potentially through targeted flavor and color adjustments to better align with consumer needs.

## Figures and Tables

**Table 1:** Influence of apple pulp on yogurt color characteristics after 1 day of storage.

Sample	L*	a*	b*	C	Hue	Sample Color ^1^
Apple pulp	40.2 ± 0.0	10.7 ± 0.0	19.1 ± 0.0	21.9 ± 0.0	60.7 ± 0.1	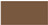
Y	92.6 ± 1.1 a	−3.9 ± 0.0 a	10.1 ± 0.2 a	10.8 ± 0.2 a	111.2 ± 0.3 d	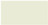
AP5Y	90.8 ± 1.2 ab	−3.1 ± 0.1 b	11.1 ± 0.1 b	11.5 ± 0.1 ab	105.6 ± 0.6 c	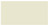
AP10Y	89.0 ± 1.2 ab	−2.2 ± 0.1 c	11.8 ± 0.2 b	12.0 ± 0.2 bc	100.4 ± 0.4 b	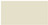
AP15Y	87.3 ± 1.2 b	−1.5 ± 0.2 d	12.7 ± 0.2 c	12.7 ± 0.2 c	96.6 ± 0.8 a	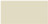
Significance	*	***	***	**	***	

^a–d^: Means within the same column for a given analysis with different lowercase superscripts differ significantly (*p* < 0.05). Y: Yogurt without apple pulp; AP5Y, AP10Y, AP15Y: Yogurts containing 5%, 10%, and 15% (*w*/*w*) apple pulp, respectively. *: *p* < 0.05; **: *p* < 0.01; and ***: *p* < 0.001. ^1^ Colors were visualized using https://colordesigner.io/convert/labtorgb (accessed on 13 June 2025).

**Table 2:** The effect of the apple pulp and storage on the textural parameters of yogurts.

Parameters	Storage (Days)	Yogurt Samples				Significance
		Y	AP5Y	AP10Y	AP15Y	
Hardness (N)	1	1.31 ± 0.10	1.35 ± 0.05	1.30 ± 0.06	1.28 ± 0.08	ns
	14	1.27 ± 0.06	1.34 ± 0.08	1.28 ± 0.07	1.25 ± 0.04	ns
	28	1.20 ± 0.08	1.31 ± 0.06	1.27 ± 0.06	1.26 ± 0.03	ns
Cohesiveness	1	0.29 ± 0.01 ^a^	0.34 ± 0.02 ^a^	0.38 ± 0.03 ^ab^	0.46 ± 0.03 ^b^	**
	14	0.29 ± 0.02 ^a^	0.36 ± 0.02 ^ab^	0.41 ± 0.01 ^b^	0.50 ± 0.02 ^c^	**
	28	0.31 ± 0.01 ^a^	0.40 ± 0.02 ^b^	0.45 ± 0.02 ^b^	0.53 ± 0.01 ^c^	***
Springiness	1	0.63 ± 0.02 ^a^	0.64 ± 0.02 ^ab^	0.72 ± 0.01 ^c^	0.70 ± 0.01 ^bc^	*
	14	0.64 ± 0.02 ^a^	0.65 ± 0.01 ^a^	0.74 ± 0.02 ^b^	0.72 ± 0.02 ^ab^	*
	28	0.66 ± 0.03 ^a^	0.66 ± 0.02 ^a^	0.78 ± 0.02 ^b^	0.76 ± 0.01 ^b^	**
Gumminess (N)	1	0.39 ± 0.04	0.38 ± 0.03	0.38 ± 0.07	0.38 ± 0.07	ns
	14	0.37 ± 0.03	0.37 ± 0.04	0.40 ± 0.01	0.43 ± 0.05	ns
	28	0.40 ± 0.03	0.39 ± 0.04	0.39 ± 0.07	0.43 ± 0.05	ns
Chewiness (N)	1	0.22 ± 0.01 ^a^	0.26 ± 0.01 ^a^	0.35 ± 0.02 ^b^	0.37 ± 0.02 ^b^	**
	14	0.24 ± 0.01 ^a^	0.27 ± 0.02 ^a^	0.36 ± 0.01 ^b^	0.34 ± 0.02 ^b^	**
	28	0.22 ± 0.02 ^a^	0.26 ± 0.01 ^a^	0.35 ± 0.01 ^b^	0.35 ± 0.01 ^b^	**

^a–c^: Means within the same row on the same day with different lowercase superscripts differ significantly (*p* < 0.05). Y: Yogurt without apple pulp; AP5Y, AP10Y, and AP15Y: Yogurts containing 5%, 10%, and 15% (*w*/*w*) apple pulp, respectively. *: *p* < 0.05; **: *p* < 0.01; and ***: *p* < 0.001; ns: not significant.

**Table 3:** Impact of storage and apple pulp on yogurt volatile compounds (semi-quantitative analysis after 14 days of storage).

Compounds (μg/kg)	Yogurt Samples ^1^			
	Y	AP5Y	AP10Y	AP15Y
**Esters**				
ethyl acetate	0.4 ± 0.2 ^a^	0.8 ± 0.2 ^ab^	1.5 ± 0.2 ^c^	1.0 ± 0.2 ^b^
ethyl butanoate	0.8 ± 0.1 ^a^	1.0 ± 0.2 ^a^	1.4 ± 0.2 ^b^	1.8 ± 0.1 ^c^
butyl acetate	Nd ^a^	Nd ^a^	0.5 ± 0.1 ^b^	0.6 ± 0.2 ^b^
2-methylbutyl acetate	Nd ^a^	0.8 ± 0.1 ^c^	0.5 ± 0.2 ^b^	0.9 ± 0.2 ^c^
ethyl octanoate	Nd ^a^	0.4 ± 0.1 ^b^	0.9 ± 0.2 ^c^	1.0 ± 0.2 ^c^
ethyl decanoate	Nd ^a^	0.5 ± 0.2 ^a^	0.4 ± 0.1 ^a^	0.9 ± 0.2 ^b^
**Total esters**	**1.2 ± 0.1 ^a^**	**3.5 ± 0.7 ^b^**	**5.2 ± 0.8 ^c^**	**6.2 ± 0.6 ^c^**
**Organic acids**				
acetic acid	10.3 ± 1.2 ^ab^	12.2 ± 1.0 ^b^	8.5 ± 0.7 ^a^	12.0 ± 0.9 ^b^
butanoic acid	5.2 ± 0.4 ^a^	10.1 ± 0.5 ^b^	9.7 ± 0.6 ^b^	10.3 ± 1.0 ^b^
hexanoic acid	20.3 ± 0.5	21.3 ± 0.8	20.5 ± 1.0	19.3 ± 1.5
octanoic acid	18.4 ± 1.0 ^ab^	17.2 ± 0.5 ^a^	19.3 ± 0.8 ^b^	18.0 ± 0.3 ^ab^
decanoic acid	15.3 ± 0.5	16.5 ± 1.0	16.0 ± 0.5	15.1 ± 0.8
**Total organic acids**	**69.5 ± 1.8 ^a^**	**77.4 ± 0.9 ^b^**	**74.0 ± 2.3 ^ab^**	**74.7 ± 2.5 ^b^**
**Alcohols**				
3-methyl-1-butanol	Nd ^a^	0.4 ± 0.2 ^ab^	1.2 ± 0.6 ^bc^	2.0 ± 0.5 ^c^
1-hexanol	4.0 ± 1.2 ^a^	5.8 ± 0.8 ^ab^	7.2 ± 0.9 ^b^	7.0 ± 0.7 ^b^
1-heptanol	5.1 ± 0.5 ^c^	4.0 ± 0.4 ^b^	1.3 ± 0.5 ^a^	1.0 ± 0.3 ^a^
1-octanol	Nd ^a^	Nd ^a^	1.2 ± 0.4 ^b^	2.8 ± 0.8 ^b^
2-heptanol	Nd ^a^	Nd ^a^	1.4 ± 0.3 ^b^	2.0 ± 0.5 ^b^
2-nonanol	1.0 ± 0.3 ^a^	1.5 ± 0.5 ^ab^	3.0 ± 0.5 ^c^	2.5 ± 0.3 ^bc^
2-ethyl-1-hexanol	2.8 ± 0.3 ^a^	7.1 ± 0.4 ^b^	12.0 ± 0.7 ^c^	10.5 ± 0.7 ^c^
**Total alcohols**	**12.9 ± 1.7 ^a^**	**18.8 ± 1.9 ^b^**	**27.3 ± 0.7 ^c^**	**28.8 ± 0.8 ^c^**
**Aldehydes**				
acetaldehyde	12.3 ± 1.2 ^ab^	10.5 ± 0.8 ^a^	13.5 ± 0.9 ^b^	14.2 ± 1.2 ^b^
hexanal	Nd ^a^	2.0 ± 0.4 ^a^	5.3 ± 0.8 ^b^	6.1 ± 1.3 ^b^
heptanal	1.5 ± 0.5	2.3 ± 0.5	2.5 ± 0.9	2.1 ± 0.5
octanal	3.2 ± 0.4	2.1 ± 0.4	3.0 ± 0.8	3.4 ± 0.7
nonanal	9.0 ± 1.3 ^a^	14.3 ± 0.9 ^b^	15.0 ± 1.2 ^b^	17.2 ± 1.4 ^b^
**Total aldehydes**	**26.0 ± 1.6 ^a^**	**31.2 ± 2.1 ^b^**	**39.3 ± 1.2 ^c^**	**43.0 ± 2.8 ^c^**
**Ketones**				
acetone	10.3 ± 0.8	8.5 ± 1.2	9.0 ± 1.3	9.5 ± 0.5
2-butanone	16.4 ± 1.0	20.3 ± 2.5	18.4 ± 1.5	18.2 ± 1.2
2-heptanone	16.3 ± 1.2	17.0 ± 0.8	16.2 ± 1.0	15.9 ± 1.2
2-octanone	9.2 ± 0.7 ^a^	14.3 ± 1.0 ^b^	13.5 ± 0.9 ^b^	15.0 ± 0.9 ^b^
**Total ketones**	**52.2 ± 1.7 ^a^**	**60.1 ± 3.4 ^b^**	**57.1 ± 1.7 ^ab^**	**58.6 ± 1.4 ^b^**
**Total compounds**	**161.7 ± 6.7 ^a^**	**190.9 ± 8.9 ^b^**	**202.9 ± 6.7 ^bc^**	**211.3 ± 7.9 ^c^**

^1^ The semi-quantitative analysis based on the concentration of the internal standard (by dividing the peak areas of the compounds of interest by the peak area of the internal standard and multiplying this ratio by the concentration of the internal standard (expressed as μg/kg of yogurt)). ^a–c^: Means within the same row with different lowercase superscripts differ significantly (*p* < 0.05). Y: Yogurt without apple pulp; AP5Y, AP10Y, and AP15Y: Yogurts containing 5%, 10%, and 15% (*w*/*w*) apple pulp, respectively. Nd: Not detected.

**Table 4:** The influence of the apple pulp incorporation on the sensory properties and acceptability of yogurt.

Sample	Appearance	Aroma	Flavor	Mouthfeel	Overall Acceptability
Y	8.5 ± 0.4 ^b^	7.6 ± 0.5	7.6 ± 0.6	7.7 ± 0.8 ^b^	7.8 ± 0.7 ^b^
AP5Y	7.5 ± 0.7 ^ab^	7.8 ± 0.7	7.0 ± 0.7	6.0 ± 0.7 ^ab^	6.7 ± 0.4 ^ab^
AP10Y	5.9 ± 0.9 ^ab^	7.5 ± 0.7	6.4 ± 0.7	4.8 ± 0.6 ^a^	5.1 ± 0.7 ^a^
AP15Y	5.3 ± 0.4 ^a^	7.9 ± 0.8	6.4 ± 0.8	4.8 ± 0.7 ^a^	5.1 ± 0.7 ^a^

^a–b^: Means within the same column with different lowercase superscripts differ significantly (*p* < 0.05). Y: Yogurt without apple pulp; AP5Y, AP10Y, and AP15Y: Yogurts containing 5%, 10%, and 15% (*w*/*w*) apple pulp, respectively.

## Data Availability

The original contributions presented in this study are included in the article. Further inquiries can be directed to the corresponding authors.

## Abbreviations

The following abbreviations are used in this manuscript:

GC/MS	Gas chromatography mass spectrometry
HS-SPME	Headspace solid-phase microextraction
Y	Yogurt without apple pulp
AP5Y	Yogurt containing 5% (*w*/*w*) apple pulp
AP10Y	Yogurt containing 10% (*w*/*w*) apple pulp
AP15Y	Yogurt containing 15% (*w*/*w*) apple pulp
